# Mitigating pharmaceutical waste exposures: policy and program considerations

**DOI:** 10.1186/s13584-016-0118-z

**Published:** 2016-11-25

**Authors:** Eric D. Amster

**Affiliations:** Department of Environmental and Occupational Health, School of Public Health, University of Haifa, Abu Hushi 199, Mount Carmel, Haifa, Israel

## Abstract

Pharmaceutical disposal and the environmental fate of medication metabolites directly impacts the public’s health in two significant ways: accidental medication ingestion of pharmaceuticals that were not disposed of properly results in inadvertent toxicity; and environmental health consequences of pharmaceuticals that were inappropriately disposed and which contaminate municipal water supply. In reviewing the effectiveness of medication disposal policy globally, it is crucial to not only determine which policies are effective but also to assess why they are effective. By assessing the root causes for a specific policy’s effectiveness it can be determined if those successes could be translated to another country with a different health care system, unique culture and divergent policy ecosystem. Any intervention regarding pharmaceutical disposal would require a multifaceted approach beyond raising awareness and coordinating pharmaceutical disposal on a national level.

While consumer participation is important, effective primary prevention would also include research on drug development that is designed to biodegrade in the environment as opposed to medications that persist and accumulate in the natural environment even when properly disposed. Countries that lack a nationalized disposal policy should leverage the resources and infrastructure already in place in the national health care system to implement a unified policy to address medication disposal in the short-term. In tandem, efforts should be made to recruit the biotechnology sector in high-tech and academia to develop new technologies in medication design and water filtration to decrease exposures in the long-term.

## Background

The matter of medication disposal has direct impact on the public’s health in two significant ways: accidental medication ingestion of pharmaceuticals that were not disposed of properly resulting in inadvertent toxicity; and environmental health consequences of pharmaceuticals that were inappropriately disposed and which contaminate the municipal water supply. In a recent IJHPR article, Barnett-Itzhaki and colleagues discuss the unique challenges of inappropriate disposal of pharmaceuticals in Israel due to the lack of any national policy or program to address the issue. To this end, the authors review national programs and policies throughout the world (focusing on Europe and North America but also noting programs in selected Middle Eastern countries) and suggest possible policy directions for Israel [1].

## Towards effective policy

In reviewing the effectiveness of medication disposal policy globally, it is crucial to not only determine which policies are effective, but also to assess why they are effective. By assessing the root causes for a specific policy’s effectiveness it can be determined if those successes could be translated to another country with a different health care system, unique culture and divergent policy ecosystem. The application of environmental public health policies from one country to another can be met with resistance and limited success. One such example is the challenge of “harmonizing” pesticide legislation worldwide when international trade organizations such as NAFTA have attempted to apply established policies from developed countries to developing nations [2]. When Mexico attempted to adopt US clean air laws in the 1990s, the aging Mexican auto fleet lacked the catalytic converters needed to run unleaded gasoline, resulting in a temporary increase in emissions. Since “copy-paste” public health policy can be inefficient at its best, and harmful at its worst, it is best to emulate the principles behind effective policies rather than attempting to copy the policy itself.

This is exceedingly true when applied to the topic of pharmaceutical waste in Israel. For example, regulations in certain states in the United States and published guidelines of the Poison Control Center recommend flushing certain pharmaceuticals down the toilet [3]. In Israel, a world-leader in water reclamation for agriculture (see below), this would result in a significant increase in pharmaceutical metabolite concentration in produce. Conversely, a pharmacy-directed medication return program such as the EnviRx program launched in British Columbia in 1996 [4], may have even greater success in Israel where most pharmacies are owned by the HMOs as opposed to small private entities. Any pharmaceutical disposal policy would need to be integrated in the national healthcare system across the four community based HMOs and governmental hospitals.

Disposal policies such as collection and destruction systems ultimately are only as successful as the public’s participation. As noted in Barnett-Itzhaki’s article, even throughout the EU, which has implemented a number of directives on the issue dating back to 2001, compliance with disposal policies is at 50% [5]. Therefore, the implementation of disposal policies is an integral component of compliance and eventual success. Health promotion strategies such as consumer marketing and advertising through traditional and new media has been effective in public health campaigns in Israel and globally.

The authors note that lack of awareness among consumers and physicians “regarding the health and environmental implications of medication accumulation and disposal” is a significant barrier in the successful application of any program [1]. While this is likely true, awareness alone is not sufficient to motivate change in individual health behavior. In applying the health belief model [6], perceived severity, susceptibility, benefits and barriers of inappropriate medication disposal would factor into the likelihood that consumers would participate in disposal programs. Even if awareness of the issue is addressed through social networks, publicity projects, campaigns and brochures as the authors advise, perceived health severity of inappropriate disposal and the perceived personal benefit of disposal program participation would likely remain low. The integrated behavior model suggests that behavior is determined by factors beyond awareness and that intention is determined by factors including norms and attitudes [7]. One of the three methods for changing social health norms is by making peer behavior visible and creating peer expectations [8]. Pharmaceutical disposal and participation in disposal programs is not a visible peer behavior and would be difficult to become a social norm through awareness alone.

## A multifaceted approach

Any intervention on this issue would require a multifaceted approach beyond raising awareness and coordinating pharmaceutical disposal on a national level. An assessment must be carried out on the natural fate of medications through their lifespan from production to disposal. This would answer the question of which medications are most likely to end up in the environment and which prescriptions present the greatest public health risk. This would enable a targeted abatement program for specific classes of medications. Medications that persist in the environment longer would also be given higher priority in a targeted abatement program. Healthcare providers and pharmacies can invest in programs for those specific medications such as automated reminders to the pharmacist at the time of medication renewal or requiring that the patient bring in the previous medication bottle or container at the time of refill. Applications on mobile devices such as pill trackers can be integrated into the care of patients with chronic illness, as well as the care of those patients who are at the highest risk of poly-pharmacy and inappropriate medication disposal.

Surveillance of policy compliance should be carried out regularly throughout the health system from multiple perspectives (HMOs, hospitals, pharmacies, consumers) and across diverse sectors of the population. Effectiveness of policy implementation should not only be viewed in terms of consumer participation; downstream effects should also be followed. For example, a natural benefit of improved medication disposal would be the decline of accidental medication ingestion monitored by the national poison control center, and decreased concentration of medication metabolites in recycled water monitored by the Ministry of Environmental Protection and local water authorities.

## Designing more ecological pharmaceuticals

The above-mentioned interventions require the involvement of multiple stake-holders and *active* participation of the health care consumer. In contrast, truly effective primary prevention would be *passive* and include research on drug development that is designed to biodegrade in the environment as opposed to medications that persist and accumulate in the natural environment even when properly disposed. This would also address the issue of medication metabolites that are excreted in urine and feces and end up in sewage treatment plants, facilities which are not designed to filter out medications and their metabolites. The efficacy of filtration systems varies with both the specific medication and the type of purification process utilized at the sewage treatment plant. Estimates vary from 65% reduction in medication concentration to 0% for some medications such as psychoactive medications, which are not filtered by sewage treatment plants [9].

In a country such as Israel which reclaims up to 90% of waste water for agricultural purposes [10], pharmaceutical waste products and metabolites inevitably end up in the food supply. Grossberger and colleagues found that certain medications such as carbamazepine, lamotrigine, caffeine, metoprolol, sulfamethoxazole and sildenafil persisted in agricultural soils irrigated with treated wastewater [11]. This suggests that even the most successful household medical waste disposal policy would leave the issue of excreted pharmaceutical metabolites unaddressed. In addition to addressing medication design, delivery and environmental fate, municipal sewage treatment facilities, specifically in areas where pharmaceutical industries operate should be equipped to address the growing issue of pharmaceuticals in the water supply.

## Addressing industrial pharmaceutical waste

As noted in Barnett-Itzhaki’s article, many governments have modeled the “polluter pays” principle, although in terms of household pharmaceutical waste it is unclear who the “polluter” is. Is the pharmaceutical industry the “polluter” or is it the health care consumer? And what role does the pharmacy and provider play beyond advocating awareness? Who is the responsible party? Ultimately, it comes down to a question of “Who should fund such programs?” These questions are difficult to answer in terms of household pharmaceutical waste; however, in terms of industrial pharmaceutical waste the question (and the answer) is much clearer. If Israeli pharmaceutical companies are sponsoring medication disposal programs in countries such as Mexico [12], it is reasonable to expect that the 20 billion dollar Israeli pharmaceutical industry will contribute to the development and implementation of programs in Israel.

In terms of quantity and potential public health impact, industrial pharmaceutical waste is a much greater issue than household pharmaceutical waste. Israeli pharmaceutical companies are the leading producers of generic medications in the world [13]. The quantity of unused, disqualified, expired medications as well us production by-product and residual that is discharged as industrial effluent is unreported, even though is likely to be significant. The high concentrations of pharmaceutical material discharged from factories far outpaces the ability of the municipal water treatment facility’s ability to treat wastewater when compared to the relatively low concentration of metabolites disposed of and excreted by the health care consumer. As industrial discharge of pharmaceuticals involves a point source exposure, it is easier to monitor and regulate than consumer disposal. This would involve a different regulatory mechanism than addressing household pharmaceutical waste and would require leadership from the Ministry of Environmental Protection on the matter.

## Conclusions

In some regards, Israel and other countries with no existing national policy or program for medication disposal are at an advantage. There is no set precedent or pretext that would limit innovation in designing novel intervention. There are no ineffective policies or inefficient systems already in place tying up national resources. There are no competing and at times contradictory policies and programs in place as is the case in the United States or Canada. Considering this and the limited success of existing national policies in other countries, countries with no current existing national policy should not rush to copy inefficient existing programs but rather should design tailor = made programs and implement policies that are appropriate to their unique health care system and public. Israel should leverage the resources and infrastructure already in place in the community based HMOs and regional medical centers to implement a unified policy to address medication disposal in the short-term. In tandem, an effort should be made to recruit the biotechnology sector in high-tech and academia to develop new technologies in medication design and water filtration to decrease exposures in the long-term.

## Abbreviations

HMO: Health Maintenance Organization
IJHPR: Israel Journal of Public Health
NAFTA: North American Free Trade Agreement
